# Draft Genome Sequence of Bacillus subtilis subsp. *subtilis* MD 32, Isolated from the Korean Fermented Food Kimchi

**DOI:** 10.1128/MRA.01376-20

**Published:** 2021-01-21

**Authors:** Ji-Young Lee, Gyu-Sung Cho, Charles M. A. P. Franz, Dae-Ook Kang

**Affiliations:** a Interdisciplinary Program in Biotechnology, Graduate School, Changwon National University, Changwon, South Korea; b Max Rubner-Institut, Federal Research Institute for Nutrition and Food, Department of Microbiology and Biotechnology, Kiel, Germany; c Department of Bio Health Science, Changwon National University, Changwon, South Korea; University of Rochester School of Medicine and Dentistry

## Abstract

Bacillus subtilis subsp. *subtilis* MD 32 was isolated from kimchi. The strain was sequenced using an Illumina MiSeq platform, and the genome size was 4,238,856 bp with a GC content of 43.41 mol%. The genome encoded 4,396 proteins, with 45 tRNAs, 6 rRNAs, and 5 noncoding RNAs.

## ANNOUNCEMENT

Bacillus subtilis is a Gram-positive, rod-shaped bacterium that can form heat-resistant spores but is not considered a human pathogen. B. subtilis has received much attention in the food industry because it is considered safe, it is involved in various fermented foods, and it produces a bacteriocin (1, 2). We conducted genome sequencing of *B. subtilis* subsp. *subtilis* MD 32 to analyze the bacteriocin gene and to facilitate the development of a biopreservative. In this study, 25 g of kimchi was blended in 225 ml of quarter-strength Ringer’s solution (Merck, Germany) for 1 min. The mixed samples were diluted with Ringer’s solution (Merck) in a 10-fold series, and 100 μl of the dilutions was plated onto LB agar and then incubated for 18 h at 37°C under aerobic conditions. A randomly picked colony was purified by streaking three times onto LB agar plates with aerobic incubation overnight at 37°C. The selected strain was presumptively identified as B. subtilis by 16S rRNA gene sequencing. The total genomic DNA of the strain was extracted from 5 ml of fresh culture grown aerobically in LB broth overnight at 37°C, with shaking at 200 rpm, using the peqGOLD bacterial DNA kit (PeqLab, Germany) according to the manufacturer’s instructions. A genomic DNA library of strain MD 32 was prepared with a Nextera XT library preparation kit (Illumina, USA), and paired-end sequencing was performed on a MiSeq sequencer (Illumina) with 2 × 250 cycles. In total, 734,725 paired-end raw sequencing reads were trimmed using the Trimmomatic (v. 0.32) pipeline (3) (with the following parameters: sliding window, 4:15; leading, 3; and minlen, 45) and then *de novo* assembled with SPAdes (v. 3.11.2) with parameters k-mer 77, careful, and minimum contig length of 500 bp (4). The quality of the draft genome sequences obtained was evaluated using the QUAST tool (5), and the *N*_50_ was 433,124 bp. All contigs were annotated using the PATRIC server and the NCBI Prokaryotic Genome Annotation Pipeline (PGAP) (v. 4.13) with default parameters (6, 7). Acquired antibiotic resistance genes were identified using the ResFinder pipeline (8), while plasmid-related sequences were detected using the PlasmidFinder pipeline (9). Except for the SPAdes pipeline, default parameters were used for all software. The draft genome sequence of strain MD 32 showed that this strain possessed 4,396 coding sequences (CDSs), 6 rRNA genes, and 45 tRNA genes, with a GC content of 43.41 mol%. Orthologous average nucleotide identity (ANI) was calculated with closely related type strains using the orthologous ANI tool (OAT) (10). The ANI values for the MD 32 strain with respect to *Bacillus* type strains B. subtilis subsp. *subtilis* KCTC 1028^T^, Bacillus licheniformis ATCC 14580^T^, and Bacillus atrophaeus NRRL NRS-213^T^ were 98.11%, 72.46%, and 79.32%, respectively. This indicated that strain MD 32 could be identified as a B. subtilis subsp. *subtilis* strain. No plasmid-related sequences and none of the typical *Bacillus* toxin genes were detected. Two antibiotic resistance genes [*mph*(*k*) and *aadk*] were present. A *sbo-alb* (*sboA* and *albA* to *albG*) operon involved in bacteriocin production could be identified, which contained the subtilosin A gene (*sboA*) and the subtilosin immunity gene (*albB*) (11). The bacteriocin gene-positive B. subtilis subsp. *subtilis* MD 32 could potentially be of use in the food and feed industries for application as a natural biopreservative (Table 1).

**TABLE 1:** *De novo* assembly of Bacillus subtilis subsp. *subtilis* MD 32 and draft genome features

Genome feature	Data
No. of contigs	54
Size of largest contig (bp)	1,215,911
*N*_50_ (bp)	433,124
GC content (mol%)	43.41
Total length (bp)	4,238,856
Genome coverage (×)	43
No. of CDSs	4,396
No. of tRNAs	45
No. of rRNAs	6
No. of noncoding RNAs	5
Bacteriocin	Subtilosin A
Methylisocitrate lyase	+^*a*^
Acquired antibiotic resistance genes	*aadk* and *mph*(*k*)

a +, detected.

### Data availability.

The draft genome sequence of Bacillus subtilis subsp. *subtilis* MD 32 was deposited in DDBJ/ENA/GenBank under the accession number JADAQW000000000. The draft genome project data have been submitted under BioProject accession number PRJNA666566 and Sequence Read Archive (SRA) accession number SRR12774112.
